# Vibration therapy in young children with mild to moderate cerebral palsy: does frequency and treatment duration matter? A randomised-controlled study

**DOI:** 10.1186/s12887-022-03786-1

**Published:** 2023-01-02

**Authors:** Alena Adaikina, José G. B. Derraik, Paul L. Hofman, Silmara Gusso

**Affiliations:** 1 Liggins Institute, University of Auckland, Auckland, New Zealand; 2 Environmental - Occupational Health Sciences and Non-Communicable Diseases Research Group, Research Institute for Health Sciences, Chiang Mai University, Chiang Mai, Thailand; 3 Department of Women’s and Children’s Health, Uppsala University, Uppsala, Sweden; 4 Exercise Sciences Department, University of Auckland, Auckland, New Zealand

**Keywords:** Cerebral palsy, Vibration therapy, Mobility, Bone mineral density, Gross motor function, Muscle function, Muscle strength, Quality of life

## Abstract

**Background:**

Vibration therapy (VT) has been increasingly studied in children with cerebral palsy (CP) over the last years, however, optimal therapeutic VT protocols are yet to be determined. The present study compared the effects of side-alternating VT protocols varying in frequency and treatment duration on the health of young children with mild-to-moderate CP.

**Methods:**

Thirty-four participants aged 6.0 to 12.6 years with CP acted as their own controls and underwent two consecutive study periods: a 12-week lead-in (control) period prior to the intervention period of 20-week side-alternating VT (9 min/session, 4 days/week), with the frequency either 20 Hz or 25 Hz, determined by randomisation. Participants had 4 assessment visits: baseline, after the control period, after 12-week VT (12VT), and after further 8 weeks of VT (20VT). Assessments included 6-minute walk test (6MWT); dual-energy x-ray absorptiometry; gross motor function; muscle function testing on the Leonardo mechanography plate and by hand-held dynamometry, and a quality-of-life questionnaire (CP QOL). Analysis was carried out using linear mixed models based on repeated measures.

**Results:**

Side-alternating VT was well-tolerated, with occasional mild itchiness reported. The median compliance level was 99%. VT led to improvements in 6MWT (+ 23 m; *p* = 0.007 after 20VT), gross motor function in standing skills (+ 0.8 points; *p* = 0.008 after 12VT; and + 1.3 points; *p* = 0.001 after 20VT) and in walking, running and jumping skills (+ 2.5 points; *p* < 0.0001 after 12VT; and + 3.7 points; p < 0.0001 after 20VT), spine bone mineral density z-score (+ 0.14; *p* = 0.015 after 20VT), velocity rise maximum of the chair rising test (+ 0.14 m/s; *p* = 0.021 after 20VT), force maximum of the single two-leg jump test (+ 0.30 N/kg; *p* = 0.0005 after 12VT; and + 0.46 N/kg; *p* = 0.022 after 20VT) and in the health module of CP QOL (+ 7 points; *p* = 0.0095 after 20VT). There were no observed differences between the two VT frequencies (i.e., 20 Hz vs 25 Hz) on study outcomes.

**Conclusions:**

The study confirms that side-alternating VT has positive effects on mobility, gross motor function, body composition, muscle function, and quality of life, independent of VT frequencies tested. Long-term, 20VT appears to be a more efficient treatment duration than a short-term, 12VT.

**Trial registration:**

Australian New Zealand Clinical Trials Registry ACTRN12618002026202; 18/12/2018.

**Supplementary Information:**

The online version contains supplementary material available at 10.1186/s12887-022-03786-1.

## Introduction

There is increasing evidence that vibration therapy (VT) is an effective rehabilitation tool for children with neuromuscular disorders, including cerebral palsy (CP). It has been found to be effective in improving mobility [1–3], muscle function [1, 2, 4] and strength [1, 5], as well as bone mineral density [2, 4, 6], gross motor function [1, 4], and quality of life [2] in children and young adults with CP.

Although VT has been increasingly studied in children with CP over the last decade, optimal therapeutic VT protocols are yet to be determined. VT protocol is defined according to the VT frequency, peak-to-peak amplitude, direction of vibration, and duration of treatment. Frequency, or the number of complete oscillation cycles per second, has been reported in the literature across a relatively wide range from 5 to 30 Hz [1–4, 7, 8]. Peak-to-peak amplitude, the maximal displacement of the oscillatory motion, has varied between 1 and 4 mm [2, 9]. According to the direction of vibration signals, VT is divided into two main types: side-alternating and vertical (synchronous) vibration mode [10]. Vibration signals in the vertical mode (vertical VT, vVT) transfer to both feet synchronously, whereas the side-alternating vibration mode (side-alternating VT, sVT) elicits the right and left leg activation alternatively [10]. In children with CP, sVT is the most used type [11], likely due to better tolerability to its vibration impulses due to reduced head vibration compared to vVT [12]. The duration of the VT program has also varied, from short-term (3 to 12 weeks) [1, 7, 9, 13] to long-term (20 to 24 weeks) [2–4]. Most of the published long-term studies investigated the VT effectiveness in adolescents or heterogeneous age groups with prepubertal and postpubertal children involved [2, 4]. In addition, these studies tended to have a small sample size and were not powered to detect changes specific to the younger age group. Moreover, to date, no longitudinal studies have been published comparing different VT protocols in young children with CP. Therefore, despite the promising results demonstrated by VT, its wider application is limited by the heterogeneity of methodological approaches, with protocols varying in frequency and duration, which hinders the interpretation of the results and the development of treatment protocols [1, 3–7, 14].

The present study sought to compare the potential effects of sVT protocols according to duration and frequency on mobility, motor function, muscle and bone health, and quality of life in young children aged 5–12 years with CP Gross Motor Function Classification System (GMFCS) level I-III.

## Materials and methods

### Participants

Participants were recruited through Starship Children’s Hospital (Auckland, NZ), Waikato Hospital (Hamilton, NZ), satellite schools, and word-of-mouth (i.e., self-referrals). Children aged 5 to 12 years 11 months with a diagnosis of any type of CP and level I-III on the GMFCS were recruited for the study. Additional inclusion criteria included the ability to understand and follow the instructions on the study protocol, ability to safely stand on a vibration plate with or without support, and having no planned surgery within 8 months before/after entering the study. The exclusion criteria included a bone fracture within 12 weeks of enrolment, history of using anabolic agents, glucocorticoids (excluding inhaled), or growth hormone therapy for at least 1 month within 3 months prior to enrolment, history of botulinum toxin injection into the lower limb(s) within 3 months before enrolment, and a history of an illness or findings on physical examination that might prevent a child from completing the study (e.g., acute thrombosis or tendinitis) [15].

### Study design

**Fig. 1:**
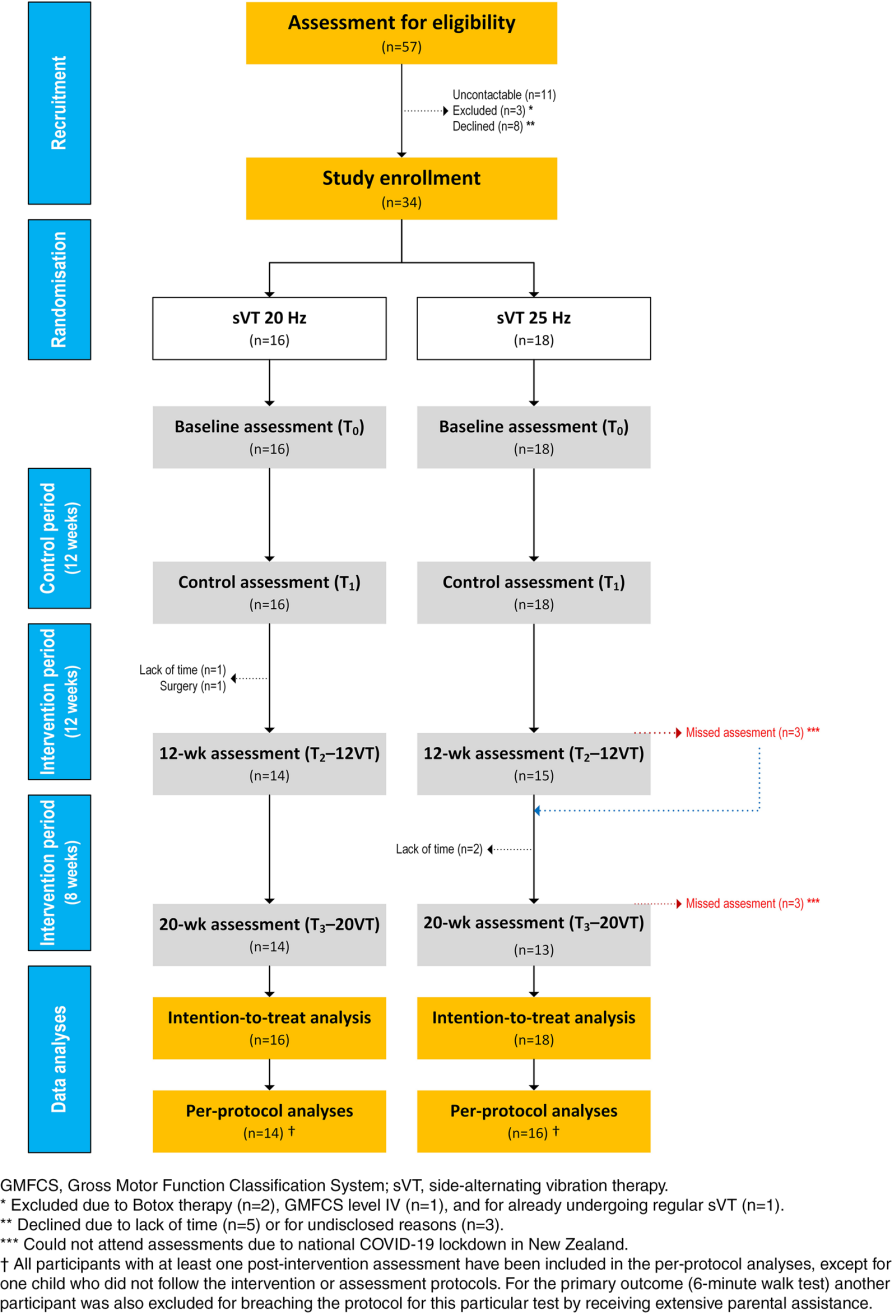
CONSORT diagram

This was a randomised, prospective interventional study with each participant acting as their own control, with 12 weeks of a lead-in control period prior to 20 weeks of intervention (Fig. 1). During their first visit, participants were randomly assigned following simple randomization procedures (computerized random number generator at https://www.randomizer.org) to one of two groups of sVT at 20 Hz or 25 Hz. Participants of both group had four assessment visits to the Maurice and Agnes Paykel Clinical Research Unit (Liggins Institute, University of Auckland) between 2019 and 2021. Following the baseline assessment (T0), participants underwent a 12-week lead-in “control” period, followed by a pre-intervention assessment (T1). Immediately after the T1 assessment, participants started a 20-week intervention period (i.e., sVT). After 12 weeks of the VT, participants had a third assessment (T2-12VT), followed by another 8 weeks of intervention and the final assessment (T3-20VT).

The study procedures were performed by the same research team members who were unblinded to participants groups. Researchers were required to know the group frequency to provide appropriate monitoring of sessions and training progression (e.g. monitor correct frequency use, increase in frequency). In addition, the vibration frequency display can be easily observed by both researchers and participants, which unblinds participants to the group allocation.

During the control period, participants continued with their usual lifestyle and were recommended to avoid starting any new activities during the study duration. Throughout the intervention period, in addition to activities during the control period, participants underwent sVT.

### Vibration therapy protocol

sVT was performed on Galileo Basic vibration plates (Novotec Medical, Pforzheim, Germany) 4 days a week, for 9 minutes at a target frequency of either 20 Hz or 25 Hz, and amplitude 2–4 mm. During the sVT sessions, participants were instructed to stand barefoot on the vibration plate with knees bent at approximately 30 degrees, with their back straight, and arms free. Families were given the option to perform sVT sessions at school or at home. School-based sVT sessions were supervised by one of the investigators (AA) and/or school physiotherapists who were familiar with sVT and participants’ supervision specifications. Home-based sVT sessions were supervised by parents/caregivers, who had an instruction session with researchers before commencing sVT. To ensure safety and monitor progress, an investigator provided regular support to families via researcher-supervised sessions at home and by contacting families via phone/email. Participants and their parents/caregivers were asked to complete a VT diary, by recording sVT sessions, reasons for missing sessions, and side effects (if any).

### Assessments

The primary outcome measure was mobility, assessed by a 6-minute walk test (6MWT). Secondary outcome measures included gross motor function, body composition, muscle strength, balance, and health-related quality of life. At the beginning of each assessment visit, participants’ anthropometry data (i.e., height, weight), blood pressure and pulse were measured as described in detail in the published study protocol [15].

#### Mobility

To assess mobility, a 6MWT, which has been shown to have good-to-excellent test-retest reliability and validity in children with CP; and is easy and safe to perform was used [16–18]. For the test, participants were instructed to walk as fast as they could for 6 minutes over the flat straight indoor corridor between two cones distanced 25 m apart [15]. The total walked distance to the nearest 0.5 m, along with the time taken to reach individual milestones (50 m) were recorded.

In addition to the 6MWT, a ten-metre walk test (10MWT) was used to assess gait speed. For this test, a straight flat indoor corridor was marked at 0, 2, 8, and 10 m [19]. Children were instructed to walk at the fastest pace from 0 to 10 m marks, and the time covered between 2 and 8 m marks was recorded. 10MWT was performed three times with a rest period of 30 seconds between trials, and the average time of three attempts was taken for speed calculation (i.e., 6 m/time in sec). The test was performed barefoot; participants were allowed to use a walking aid.

#### Gross motor function

Gross motor function was evaluated using the Gross Motor Function Measure-88 (GMFM-88), a reliable and valid scale for applied research in children with CP [20, 21]. The GMFM-88 (88 items) is standardized for use in children aged between 5 months and 16 years and is divided into 5 dimensions: (A) lying/rolling, (B) sitting, (C) crawling/kneeling, (D) standing, and (E) walking/running/jumping [22]. In this study, we assessed dimensions D (GMFM-D) and E (GMFM-E).

#### Body composition

Whole-body and lumbar spine (L1-L4) dual-energy X-ray absorptiometry scans (Lunar iDXA, GE Healthcare, Madison, WI, USA) were performed to measure body composition. These two sites are recommended by the International Society for Clinical Densitometry as the most accurate and reproducible sites in children to assess bone mineral density [23]. Key parameters of interest included total body less head (TBLH), areal bone mineral density (aBMD), bone mineral content (BMC), lean mass, and fat mass.

#### Muscle function

Muscle function was assessed by a hand-held dynamometry (HHD), the chair rising test (CRT), the single two-leg jump test (STLJT), and the balance test (BT). Muscle strength of five muscle groups in both legs was assessed with an HHD (MicroFET2, Hoggan Scientific, USA) by a “make” technique [24]. This included hip flexors and extensors, knee flexors and extensors, and ankle dorsiflexors. Muscle strength was measured three times on each leg, and the average was used for analysis. CRT, STLJT, and double leg BT were performed on the Leonardo™ Mechanography Ground Reaction Force Plate (Novotec Medical, Pforzheim, Germany), a reliable and valid instrument in children with musculoskeletal disabilities including CP [2, 25, 26]. Each test was performed three times, and the best result was recorded for analysis: CRT – the fastest time to complete the test; STLJ – the maximum peak velocity; double leg BT – the smallest elliptical area [15, 25].

#### Health-related quality of life

The Cerebral Palsy Quality of Life Questionnaire for Primary Caregiver (CP QOL) was administered to evaluate participants’ well-being, participation, communication, pain and feelings about disability, and family health. The questionnaire has strong validity and reliability [27] and is widely used for research purposes [2, 28]. During each assessment visit, the questionnaire was filled out by the same person (a parent or a caregiver) to avoid a different perception of a child’s well-being. The total score for each domain was calculated and analysed.

Please note, that due to variations in CP presentation (i.e., GMFCS level), some assessments were not performed by all participants; the respective number is reported in the tables with outcomes.

### Statistical analyses

The sample size calculation is described in the published study ptotocol [15].

The potential effects of sVT on the primary outcome (6MWT) were performed based on intention-to-treat (ITT), including all data recorded throughout the trial. Per-protocol analyses (PPA) were also run on the primary outcome and all secondary outcomes, excluding data associated with protocol violations.

Analyses were carried out using linear mixed models based on repeated measures. The model for any given outcome included the three sequential measurements (if available) for all participants at the end of the Control period and after 12 weeks and 20 weeks of sVT (12VT and 20VT, respectively). Models included the study ID as a random factor to account for the non-independence of multiple measurements on the same participant, study period (i.e., Control, 12VT, and 20VT), and randomisation group (20 vs 25 Hz), with participant’s GMFCS level and the value of the outcome at baseline (T0) also included as covariates.

In addition, the linear association between the baseline values for a given outcome and the participants’ ages at baseline were assessed using Pearson’s correlation coefficients; where a statistically significant association was observed (at *p* < 0.05), the number of days elapsed between the baseline assessment and a given follow-up assessment was also included as a covariate to account for the participants’ potential linear growth throughout the study.

Potential 2-way and 3-way interactions between the study period, randomisation group, and GMFCS level were assessed for all models, and where a significant interaction was present results were reported accordingly. However, non-significant interactions were removed from the models.

Data are reported as the least-squares means (i.e., adjusted means) and respective 95% confidence intervals (CI), with pairwise differences between assessments reported as the adjusted mean differences (aMD) and the 95% CI. Compliance data are reported as the median, quartile 1 (Q1, 25th percentile), and quartile 3 (Q3, 75th percentile). The distribution of all outcomes was examined, and, where appropriate, data were log-transformed to approximate a normal distribution, with results back-transformed for reporting.

Data were analysed using SAS v9.4 (SAS Institute, Cary, NC, USA). There was no imputation of missing values. All statistical tests were two-sided, with statistical significance maintained at *p* < 0.05 without adjustment for multiple comparisons as per Rothman 1990 [29].

## Results

### Study population

In total, 34 children aged 6.0 to 12.6 years were enrolled in the study, with 16 and 18 participants randomised into 20 Hz and 25 Hz groups, respectively (Fig. 1). The demographic characteristics of the study population are presented in Table 1. Four participants withdrew from the study (Fig. 1): one soon after the control assessment for having a semi-elective surgery scheduled; one after 8 weeks and two after 12 weeks of VT due to lack of time to perform the sessions. Please note that the study was partially conducted while New Zealand was under COVID-19 lockdown restrictions [30]. This scenario markedly impacted our ability to recruit participants and perform the assessments, also affecting the ability of some participants to undergo sVT in school settings. Three participants completed 20 weeks of sVT but were unable to attend the final 20VT assessment (T3).

**Table 1:** Characteristics of study participants

Parameters		Group 20 Hz	Group 25 Hz
**n**		16	18
**Age (years)**		9.5 [4.5, 11.7]	9.2 [6.9, 10.4]
**Sex**	**Females**	6 (37%)	7 (39%)
**Ethnicity**	**NZ European**	13 (81%)	11 (61%)
	**Māori**	2 (13%)	4 (22%)
	**Other**	1 (6%)	3 (17%)
**GMFCS**	**Level I**	6 (37%)	7 (39%)
	**Level II**	7 (44%)	8 (44%)
	**Level III**	3 (19%)	3 (17%)
**CP type**	**Spastic**	13 (81%)	13 (72%)
	**Dystonic**	3 (19%)	2 (11%)
	**Ataxic**	nil	1 (6%)
	**Unknown**	nil	2 (11%)

*CP* Cerebral palsy; *GMFCS* Gross Motor Function Classification System

Age data are median [quartile 1, quartile 3] and categorical data are n (%)

During the study, four children underwent regular home-based physiotherapy once or twice a week with a session duration from 20 to 60 min. They were conducted before the study commenced and throughout their participation (i.e., during control and intervention periods). Therefore, no additional activities were implemented during the study duration.

### Effects of sVT frequency and duration

There were no observed differences between the two sVT frequencies (i.e., 20 Hz vs 25 Hz) on study outcomes, but there were some differences associated with sVT duration (i.e., 12 vs 20 weeks). As a result, study outcomes are reported for the overall pairwise differences (Control period vs 12VT and Control vs 20VT), except for outcomes with an observed effect of sVT duration, for which differences between 12VT and 20VT are also reported.

### Mobility (primary outcome)

**Fig. 2:**
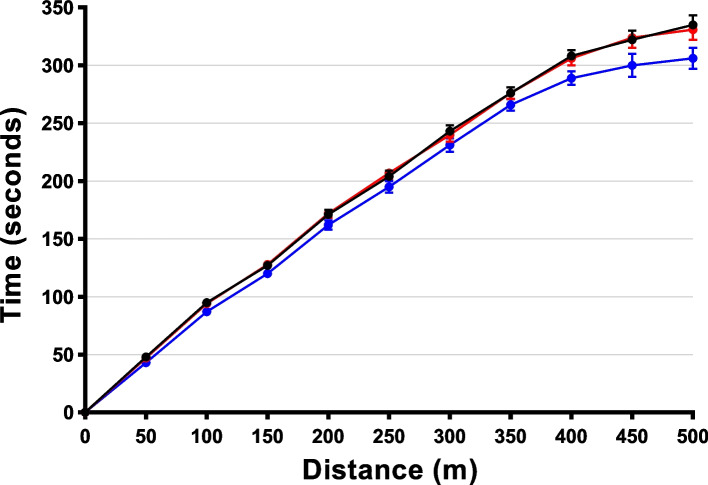
Time taken to reach distance milestones in the 6-minute walk test among children with cerebral palsy after the Control period (black) and after side-alternating vibration therapy for 12 weeks (red) and 20 weeks (blue)

The results of mobility outcomes are presented in Table 2. For the primary outcome, 20VT (but not 12VT) led to improvements in the 6MWT, with participants covering additional 23 m according to both ITT (*p* = 0.007) and PPA (*p* = 0.011) analyses (Table 2), with distance milestones reached progressively faster (Fig. 2). Participants also showed improvements in the 10MWT (Table 2), with an increase of 0.18 m/s in gait speed after 20VT (*p* = 0.047).

**Table 2:** Mobility parameters outcomes

Parameters	n	Control period	12VT	20VT	12VT vs Control	20VT vs Control	20VT vs 12VT
**6MWT ITT (m)**	34	406 (391, 422)	407 (391, 423)	429 (413, 446)	0 (− 12, 13)	23 (6, 39)**	**22 (9, 36)**^**††**^
**6MWT PPA (m)**	29	421 (404, 438)	422 (405, 439)	444 (427, 462)	1 (−12, 15)	23 (6, 41)*	**22 (8, 37)**^**††**^
**10MWT (m/s)**	25	2.27 (2.12, 2.42)	2.36 (2.21, 2.51)	2.46 (2.29, 2.62)	0.09 (− 0.05, 0.26)	0.18 (0.00, 0.37)*	0.10 (− 0.06, 0.25)

6*MWT* 6-minute walk test; 10*MWT* 10-m walk test; 12*VT* assessment after 12 weeks of side-alternating vibration therapy; 20*VT* assessment after 20 weeks of side-alternating vibration therapy; *ITT* intention-to-treat analysis; *PPA* per-protocol analysis

Data at each assessment are the adjusted means and 95% confidence intervals (CI), while differences between assessments are the adjusted mean differences and 95% CI; all values were derived from linear mixed models based on repeated measures including the participant’s GMFCS level, randomisation group (20 Hz / 25 Hz), and the baseline value of the outcome as a covariate

*p*-values for statistically significant differences (at *p* < 0.05) between two given assessments are shown in bold; *p < 0.05 and **p < 0.01 for pairwise differences compared to the Control period; ^††^*p* < 0.01 for a difference between 12VT and 20VT

*n* is the number of participants at baseline; the number of participants who completed a given assessment is provided in Additional file 1

### Gross motor function

Both 12VT and 20VT led to improvements in gross motor function, measured by both GMFM-D and GMFM-E (Table 3). GMFM-E scores improved by 2.5 points after 12VT (+ 3.5%; 95% CI 2.3, 4.7%; *p* < 0.0001) and by 3.7 points after 20VT (+ 5.1%; 95% CI 3.6, 6.6%; *p* < 0.0001) (Table 3). For GMFM-D, there was a significant interaction between GMFCS level and assessment (*p* = 0.0009), indicating a differential response to sVT. Among participants with GMFCS level I and II, GMFM-D scores increased by 0.8 points after 12VT (+ 2.1%; 95% CI 0.6, 3.6%; *p* = 0.008) and by 1.3 points after 20VT (+ 3.4%; 95% CI 1.4, 5.4%; *p* = 0.001) (Table 3). There was a greater increase in GMFM-D scores among children with GMFCS level III of 3.0 points after 12VT (+ 7.7%; 95% CI 4.1, 11.3%; p < 0.0001) and 5.0 points after 20VT (+ 12.8%; 95% CI 8.5, 17.1%; p < 0.0001) (Table 3).

**Table 3:** Gross motor function outcomes

Parameters, units	n	Control period	12VT	20VT	12VT vs Control	20VT vs Control	20 VT vs 12VT
**GMFM-D (level I-II), score**	25	32.8 (31.9, 33.7)	33.6 (32.7, 34.5)	34.1 (33.2, 35.0)	0.8 (0.2, 1.4)**	1.3 (0.5, 2.1)**	0.5 (−0.1, 1.2)
**GMFM-D (level III), score**	5	26.7 (23.4, 30.1)	29.7 (26.4, 33.1)	31.7 (28.4, 35.1)	3.0 (1.6, 4.4)****	5.0 (3.3, 6.7)****	**2.0 (0.6, 3.4)**^**††**^
**GMFM-E, score**	30	49.2 (47.6, 50.8)	51.7 (50.1, 53.3)	52.9 (51.3, 54.5)	2.5 (1.6, 3.3)****	3.7 (2.6, 4.8)****	**1.2 (0.3, 2.2)**^**†**^

12VT, assessment after 12 weeks of side-alternating vibration therapy; 20VT, assessment after 20 weeks of side-alternatingvibration therapy; GMFM-D, gross motor function measure dimension D; GMFM-E, gross motor function measure dimension E

Data at each assessment are the adjusted means and 95% confidence intervals (CI), while differences between assessments are the adjusted mean differences and 95% CI; GMFM-E values were derived from linear mixed models based on repeated measures including the participant’s GMFCS level, randomisation group (20 Hz / 25 Hz), and the baseline value of the outcome as a covariate; GMFM-D values were similarly derived but with the inclusion of an interaction term between GMFCS level and assessment

Statistically significant differences (at *p* < 0.05) between assessments are shown in bold; *p < 0.05, **p < 0.01 and ****p < 0.0001 for pairwise differences compared to the Control period; ^†^p < 0.05 and ^††^p < 0.01 for differences between 12VT and 20VT

After 20VT, the extra 8 weeks of sVT lead to an additional 1.2-point increase in GMFM-E scores (+ 1.6%; 95% CI 0.3, 2.9%; *p* = 0.011), as well as a 2.0-point increase in GMFM-D scores for participants with GMFCS level III (+ 5.1%; 95% CI 1.5, 8.7%; *p* = 0.006) (Table 3).

### Body composition

sVT led to no observed changes in anthropometry (i.e., height, weight, and BMI z-scores), lean mass, or fat mass (Table 4). In contrast, spine aBMD z-scores increased by 0.14 after 20VT (*p* = 0.015), with a 1.5-g improvement also seen in spine BMC (L1-L4) after 12VT (*p* = 0.046) that was not detected after 20VT (*p* = 0.09) (Table 4). There were no observed changes in TBLH aBMD, TBLH BMC, or spine aBMD (Table 4).

**Table 4:** Anthropometry and body composition outcomes

Parameters (units)		n	Control period	12VT	20VT	12VT vs Control	20VT vs Control
**Anthropometry**	Height (z-score)	30	−0.63 (− 0.72, − 0.55)	−0.60 (− 0.69, − 0.52)	−0.58 (− 0.67, − 0.49)	0.03 (− 0.05, 0.12)	0.06 (− 0.05, 0.16)
	Weight (z-score)	30	−0.50 (− 0.60, − 0.39)	−0.51 (− 0.62, − 0.40)	−0.40 (− 0.52, − 0.29)	−0.02 (− 0.14, 0.10)	0.10 (− 0.05, 0.24)
	BMI (z-score)	30	−0.19 (− 0.35, − 0.03)	−0.23 (− 0.40, − 0.06)	−0.11 (− 0.28, 0.07)	−0.04 (− 0.25, 0.17)	0.08 (− 0.15, 0.32)
**aBMD**	TBLH (g/cm^2^)	30	0.631 (0.612, 0.650)	0.633 (0.624, 0.641)	0.633 (0.614, 0.651)	0.002 (−0.018, 0.022)	0.002 (−0.033, 0.036)
	TBLH (z-score)	30	−0.97 (−1.06, − 0.89)	−0.94 (− 1.02, − 0.85)	−0.93 (− 1.01, − 0.84)	0.03 (− 0.03, 0.11)	0.05 (− 0.05, 0.14)
	Spine L1-L4 (g/cm^2^)	29	0.685 (0.662, 0.707)	0.691 (0.682, 0.701)	0.699 (0.676, 0.723)	0.007 (−0.018, 0.031)	0.015 (− 0.029, 0.058)
	Spine L1-L4 (z-score)	27	−0.71 (− 0.79, − 0.63)	−0.63 (− 0.72, − 0.54)	−0.57 (− 0.66, − 0.48)	0.08 (− 0.02, 0.18)	0.14 (0.03, 0.26)*
**BMC**	TBLH (g)	30	741 (714, 768)	763 (749, 776)	772 (745, 800)	21 (−7, 50)	31 (−18, 81)
	Spine L1-L4 (g)	29	21.7 (20.4, 23.0)	23.2 (22.6, 23.7)	23.9 (22.5, 25.2)	1.5 (0.0, 2.9)*	2.2 (−0.3, 4.7)
**Lean mass**	Legs (kg)	30	5.9 (5.6, 6.3)	6.0 (5.9, 6.2)	6.1 (5.8, 6.5)	0.1 (−0.3, 0.4)	0.2 (−0.5, 0.8)
**Fat mass**	Total (%)	30	28.6 (28.3, 28.9)	28.8 (28.5, 29.2)	28.8 (28.4, 29.2)	0.2 (−0.3, 0.7)	0.2 (−0.3, 0.7)

12VT, assessment after 12 weeks of side-alternating vibration therapy; 20VT, assessment after 20 weeks of side-alternating vibration therapy; aBMD, areal bone mineral density; BMC, bone mineral content; BMI, body mass index; TBLH, total body less head

Data at each assessment are the adjusted means and 95% confidence intervals (CI), while differences between assessments are the adjusted mean differences and 95% CI; all values were derived from linear mixed models based on repeated measures including the participant’s GMFCS level, randomisation group (20 Hz / 25 Hz), the baseline value of the outcome, as well as the number of days elapsed from baseline (except for anthropometric outcomes)

*n* is the number of participants at baseline; the number of participants who completed a given assessment is provided in Additional file 1

Statistically significant differences (at *p* < 0.05) between assessments are shown in bold, where *p < 0.05 indicates a pairwise difference compared to the Control period

### Muscle function

Tests on the Leonardo Mechanography plate showed that sVT improved some parameters of muscle function (Table 5). The maximum velocity rise in the chair-rise test increased by 0.14 m/s (≈17%) after 20VT (*p* = 0.021; Table 5). The maximum force in the single two-leg jump test increased by 0.30 N/kg after 12VT (*p* = 0.0005), with eight extra weeks of sVT leading to an additional 0.15 N/kg improvement (95% CI 0.02, 0.28 N/kg; *p* = 0.022), so that force increased by 0.46 N/kg after 20VT (p = 0.022) (Table 5). However, there were no observed changes in double-leg balance (Table 5), or in muscle strength measured by HHD (Additional file 2).

**Table 5:** Muscle function and physical activity outcomes

Parameters (units)		n	Control	12VT	20VT	12VT vs Control	20VT vs Control
**CRT**	Force_max_ (N/kg)	8	0.52 (−0.21, 1.25)	0.71 (0.49, 0.93)	0.82 (0.04, 1.61)	0.19 (−0.71, 1.09)	0.30 (− 1.21,1.82)
	Velocity rise _max_ (m/s)	8	0.83 (0.76, 0.90)	0.89 (0.80, 0.98)	0.97 (0.89, 1.06)	0.06 (−0.06, 0.18)	0.14 (0.03, 0.26)*
**STLJT**	Force_max,_ (N/kg)	19	0.43 (0.29, 0.57)	0.74 (0.67, 0.80)	0.89 (0.75, 1.03)	0.30 (0.14, 0.47)***	0.46 (0.19, 0.73)**
	Velocity_max_ (m/s)	19	1.46 (1.30, 1.63)	1.42 (1.26, 1.59)	1.46 (1.29, 1.62)	−0.04 (−0.13, 0.05)	−0.01 (− 0.13, 0.12)
**Double leg balance**	Elliptical area (cm^2^)	26	1.38 (1.07, 1.79)	1.34 (1.02, 1.75)	1.18 (0.90, 1.56)	0.97 (0.74, 1.27)	0.86 (0.61, 1.20)

12VT, assessment after 12 weeks of side-alternating vibration therapy; 20VT, assessment after 20 side-alternating weeks of vibration therapy; CRT, chair rising test; STLJT, Single two-leg jump test

Data at each assessment are the adjusted means and 95% confidence intervals (CI), while differences between assessments are the adjusted mean differences (aMD) and 95% CI; all values were derived from linear mixed models based on repeated measures including the participant’s GMFCS level, randomisation group (20 Hz / 25 Hz), and the baseline value of the outcome, as well as the number of days elapsed from baseline for CRT Force_max_. Note the values for double-leg balance were log-transformed for analyses and back-transformed for reporting in this table, so the aMD represents the proportional difference compared to the Control period

*n* is the number of participants at baseline; the number of participants who completed a given assessment is provided in Additional file 1

Statistically significant differences (at *p* < 0.05) between assessments are shown in bold; *p < 0.05, **p < 0.01, and *** *p* < 0.001 for pairwise differences compared to the Control period

### Health-related quality of life outcomes

CPQOL was filled out by parents or caregivers of 25 participants at all assessments (Additional file 1). After 20VT, there was a 7-point improvement in general health scores (95% CI 2, 12; *p* = 0.0095), but there were no other observed effects of sVT on health-related quality of life (Additional file 3).

### Compliance and side effects

Overall, participants had a high level of compliance with the prescribed VT protocol, after both 12 weeks [median = 99% (Q1 = 92%, Q3 = 100%)] and 20 weeks [median = 99% (Q1 = 89%, Q3 = 100%)]. Only 5 out of 32 (16%) and 6 out of 30 (20%) participants who attended the 12-week and 20-week assessments, respectively, completed less than 80% of prescribed sVT sessions. The main reported reasons for missing sessions were lack of time and being unwell. Parents/caregivers of participants who performed sessions at home highlighted a higher need for child encouragement after 12 weeks of sVT, as participants appeared to be gradually losing interest in performing sVT at home. Notably, 50% of the participants were undergoing sVT during COVID-19 lockdowns, which might have also changed their level of routine activities, and impacted their mental health and behaviour. In turn, these might have negatively affected their motivation to continue sVT [31].

sVT was well-tolerated with no severe adverse events reported. Nine participants (30%) reported occasional mild itchiness in the calf and ankle areas during sVT sessions, which resolved within approximately 30 seconds to 2 minutes after cessation of the sVT session. Two of these participants also complained about occasional warmth and redness of the skin on ankle area quickly resolved after the sVT session.

## Discussion

This study is the first clinical trial comparing the effectiveness of different sVT protocols varying in duration and frequency in children with mild to moderate CP. The results demonstrated that 20 weeks of sVT are more effective in impacting walking mobility, gross motor function, and muscle function, but there were no observed differences in study outcomes between the two sVT frequencies (20 Hz vs 25 Hz). The specific underlying mechanism of the effects of VT remains unclear. However, proposed mechanisms include activation of the a-motoneurons [32] and Golgi tendinous organs [33], stimulation of the proprioceptive sensory system and secretion of hormones (e.g., growth hormone, testosterone) [32, 34]. In addition, vibration may stimulate spinal and supraspinal functions, leading to better nervous control of muscular fibre recruitment [34]. These mechanisms may allow for greater musculoskeletal system activation in individuals with limited ability to perform weight-bearing activity, providing a possible avenue through which muscle function and mobility can be increased. A positive effect of sVT on gross motor skills could be plausibly explained by the impact of vibration stimuli on the central nervous system via proprioceptive pathways, given that proprioception is an important component of motor control [35].

Long-term sVT (i.e., 20 weeks) had a positive effect on mobility in young children with CP and was more efficacious than short-term sVT. These mobility improvements were supported by 10MWT results, demonstrating an increase in gait speed after 12 weeks of sVT, with further improvements after 20 weeks (2.27 m/s → 2.36 m/s → 2.46 m/s). Our results are consistent with the findings of Gusso et al.’s study that reported improvements in 6MWT after 20 weeks of sVT in adolescents and young adults 11–21 years of age with CP GMFCS level II-IV [2]. Several studies have reported a positive effect of shorter-term VT (4 to 12 weeks) on mobility in young children with CP [1, 9, 36]. However, in those studies, VT was delivered in conjunction with stretch [9] or physiotherapy programs [1, 36], hindering reliable comparisons to our study.

There were also beneficial effects of both short-term (12 weeks) and long-term (20 weeks) sVT on gross motor function, although the longer intervention led to additional improvements beyond the 12-week gains. Our findings are important because they show the effectiveness of sVT as a single intervention, whereas previous studies have reported improvements in gross motor function after short- [1, 13, 37] and long-term VT [4] in conjunction with physiotherapy programs. Stark et al. reported 12.7% increase in GMFM-66 scores after 6 months of home-based sVT in conjunction with physiotherapy in 78 participants with spastic GMFCS I-V aged 2–24 years [4]. A recent study by Tekin and Kavlak showed that 8 weeks of vertical VT in combination with physiotherapy improved the GMFM-88 total score and dimension E (i.e., walking, running, and jumping skills) score in 11 participants aged 6–18 years with CP [37].

Given children affected by CP who have better mobility and physical function experience less restrictions in activities of daily living and social participation [38–40], we speculate that VT has the potential to improve their quality of life and social participation. This is supported by our observed improvements in the general health module and an upward tendency in the score of the communication module. Gusso et al. reported similar improvements in general well-being, participation, and school well-being after 20 weeks of sVT in 40 adolescents and young adults with CP GMFCS level II-III [2].

The Leonardo mechanography data showed an increase in maximum force measured by STLJT after 12 and 20 weeks, and maximum velocity rise measured by the CRT after 20 weeks of sVT. Conversely, we found no changes in muscle strength, in contrast to previous studies on young children with CP aged 8–12 years that reported increases in knee extension muscle strength after 12 weeks of sVT at 12–18 Hz [1, 5]. However, in those studies, sVT was delivered in conjunction with physiotherapy, and its frequency was lower than in our study. More studies are needed to explore the effect of VT on muscle strength, including the most optimal VT frequency.

As for body composition, there were improvements in spine BMD Z-score after 20 weeks of sVT and in BMC after 12 weeks, with no changes observed in TBLH and leg parameters. To date, previous studies have reported conflicting findings on the effectiveness of VT on BMD and BMC as measured by DXA. El-Bagalaty & Ismaeel reported an increase in the lumbar spine and femoral neck BMD after 12 weeks of sVT in combination with physiotherapy among 46 children aged 5–7 years with spastic CP [41]. Similarly, Stark et al. found improvements in total body BMD and BMC after 6 months of sVT administered in conjunction with intensive physiotherapy in 78 subjects aged 2–24 years [4]. However, Ruck et al. found no changes in BMD in the lumbar spine and distal femur in 10 children (mean age 8.3 years) with CP GMFCS II-IV, who had undergone 6 months of sVT [3]. Still, these findings should be interpreted with caution. Firstly, El-Bagalaty & Ismaeel [41] and Stark et al.’s studies [4] utilised sVT in combination with physiotherapy, making it impossible to isolate the effects of VT itself. Secondly, Ruck et al. study [3] had a small sample of 10 participants, which limited the ability to compare the results. These factors, taken together, indicate the need for more studies to evaluate VT’s effectiveness on bone health.

The main limitation of our study was the smaller number of participants recruited compared to our original target due to the government-imposed lockdowns associated with the COVID-19 pandemic, which also prevented some participants from completing all clinical assessments; in turn, these most likely affected our ability to detect statistically significant treatment effects on secondary outcomes.

## Conclusion

In conclusion, this study confirms that sVT led to positive effects on mobility, gross motor function, muscle function, and quality of life, independent of sVT frequency (20 Hz or 25 Hz). Long-term, 20-week sVT appears to be a more efficient treatment duration than a short-term, 12-week sVT. Nonetheless, it still remains to be determined whether VT impacts muscle strength and bone health in young children with CP. Thus, further studies investigating this effect and the impact of VT as a single intervention or in conjunction with other types of physiotherapy are required.

## Supplementary Information

**Additional file 1.** Number of participants completed the clinical assessments at each time-point.

**Additional file 2.** Muscle strength outcomes.

**Additional file 3.** Health-related quality of life outcomes.

## Data Availability

The dataset on which this study was based can be made available from the corresponding author on reasonable request, and after the necessary ethics approval is obtained.

## Abbreviations

10MWT: 10 m walk test
6MWT: 6-minute walk test
aBMD: Areal bone mineral density
aMD: Adjusted mean differences
BMC: Bone mineral content
BT: Balance test
CI: Confidence interval
CP: Cerebral palsy
CP QOL: Cerebral Palsy Quality of Life Questionnaire
CRT: Chair rising test
DXA: Dual-energy X-ray absorptiometry
GMFCS: Gross Motor Function Classification System
GMFM-88: Gross Motor Function Measure-88
GMFM-D: Gross Motor Function Measure dimension D
GMFM-E: Gross Motor Function Measure dimension E
HHD: Hand-held dynamometry
ITT: Intention-to-treat
PPA: Per-protocol analyses
STLJT: Single two-leg jump test
sVT: Side-alternating vibration therapy
TBLH: Total body less head
VT: Vibration therapy
vVT: Vertical vibration therapy
