# Supplementary material for: Vibration therapy in young children with mild to moderate cerebral palsy: does frequency and treatment duration matter? A randomised-controlled study
Source: BMC Pediatr. 2023 Jan 2;23:4. doi: 10.1186/s12887-022-03786-1 (PMC9806818; doi:10.1186/s12887-022-03786-1)
Supplement: Supplementary file 1 — Additional file 1. Number of participants completed the clinical assessments at each time-point. [file 12887_2022_3786_MOESM1_ESM.pdf]

Additional file 1

Number of participants who completed the clinical assessments at each time-point.

PARAMETERS	BASELINE	CONTROL	12VT	20VT
**6-minute walk test (intention-to-treat)**	34	34	29	27
**6-minute walk test (per-protocol analysis)**	29	29	27	25
**10-meter walk test**	25	25	22	19
**GMFM-D (level I-II)**	25	25	25	21
**GMFM-D (level III)**	5	5	4	5
**GMFM-E**	30	30	29	26
**Anthropometry** [1]	30	30	28	26
**Total body less head** [2]	30	30	28	26
**Spine L1-L4** [2]	29	29	27	24
**Spine L1-L4 Z-score**	27	25	21	20
**Legs lean mass**	30	30	28	26
**Fat mass total**	30	30	28	26
**Chair-rising test** [3]	8	8	7	7
**Single two-leg jump test** [3]	19	19	17	17
**Balance**	26	26	23	22
**Ankle dorsiflexion** [4]	19	19	17	17
**Knee flexion** [4]	18	18	17	15
**Knee extension** [4]	29	29	28	24
**Hip flexion** [4]	20	21	19	15
**Hip extension** [4]	9	9	8	6
**Quality of life questionnaire** [5]	28	28	26	25

12VT, assessment after 12 weeks of side-alternating vibration therapy; 20VT, assessment after 20 weeks of side-alternating vibration therapy; GMFM-D, gross motor function measure dimension D; GMFM-E, gross motor function measure dimension E.
[1] Height Z-score, weight Z-score, and body mass index Z-score.
[2] Areal bone mineral density and TBLH bone mineral content.
[3] $Force_{max}$ and velocity $rise_{max}$.
[4] Both dominant and non-dominant legs.
[5] Health, friends and family, communication, pain and bother, participation, and parents’ health modules.
