# Supplementary material for: Vibration therapy in young children with mild to moderate cerebral palsy: does frequency and treatment duration matter? A randomised-controlled study
Source: BMC Pediatr. 2023 Jan 2;23:4. doi: 10.1186/s12887-022-03786-1 (PMC9806818; doi:10.1186/s12887-022-03786-1)
Supplement: Supplementary file 2 — Additional file 2. Muscle strength outcomes. [file 12887_2022_3786_MOESM2_ESM.pdf]

Additional file 2

Muscle strength outcomes

Parameter	Limb	*n*	Control	12VT	20VT	12VT vs Control	20VT vs Control
**Ankle dorsiflexion**	**Dominant**	20	3.0 (1.9, 4.1)	3.1 (2.7, 3.4)	2.8 (1.7, 3.9)	0.0 (-1.2, 1.3)	-0.2 (-2.4, 1.9)
	**Non-dominant**	19	2.6 (1.6, 3.5)	2.4 (2.1, 2.7)	2.4 (1.4, 3.4)	-0.2 (-1.3, 0.9)	-0.2 (-2.0, 1.7)
**Knee flexion**	**Dominant**	18	3.2 (2.1, 4.3)	3.4 (3.1, 3.8)	4.1 (3.0, 5.3)	0.2 (-1.0, 1.4)	0.9 (-1.2, 3.0)
	**Non-dominant**	18	3.0 (1.9, 4.0)	3.4 (3.1, 3.8)	3.6 (2.6, 4.7)	0.5 (-0.7, 1.7)	0.7 (-1.3, 2.7)
**Knee extension**	**Dominant**	29	6.1 (4.9, 7.4)	6.9 (6.3, 7.4)	7.3 (6.0, 8.6)	0.7 (-0.7, 2.2)	1.1 (-1.3, 3.6)
	**Non-dominant**	29	5.5 (4.4, 6.6)	6.2 (5.7, 6.6)	6.7 (5.6, 7.9)	0.6 (-0.6, 1.9)	1.2 (-0.9, 3.3)
**Hip flexion**	**Dominant**	20	7.3 (5.7, 9.0)	6.3 (5.7, 6.9)	5.4 (3.6, 7.3)	-1.1 (-3.1, 0.9)	-1.9 (-5.3, 1.5)
	**Non-dominant**	20	6.5 (4.7, 8.2)	6.4 (5.7, 7.0)	6.3 (4.3, 8.3)	-0.1 (-2.2, 2.1)	-0.2 (-3.8, 3.5)
**Hip extension**	**Dominant**	9	4.6 (1.7, 7.6)	6.1 (5.0, 7.3)	6.8 (3.6, 10.0)	1.5 (-2.2, 5.2)	2.2 (-3.8, 8.2)
	**Non-dominant**	9	6.3 (4.0, 8.7)	5.6 (4.7, 6.5)	5.1 (2.5, 7.7)	-0.7 (-3.7, 2.3)	-1.2 (-6.1, 3.6)

12VT, assessment after 12 weeks of side-alternating vibration therapy; 20VT, assessment after 20 weeks of side-alternating vibration therapy.
Data at each assessment are the adjusted means and 95% confidence intervals (CI), while differences between assessments are the adjusted mean differences and 95% CI; all values were derived from linear mixed models based on repeated measures, adjusted for the participant's GMFCS level, group allocation (20 Hz / 25Hz), baseline value of the outcome, and the number of days elapsed from baseline.
n is the number of participants at baseline; the number of participants who completed a given assessment is provided in Additional file 1.
