# Supplementary material for: Vibration therapy in young children with mild to moderate cerebral palsy: does frequency and treatment duration matter? A randomised-controlled study
Source: BMC Pediatr. 2023 Jan 2;23:4. doi: 10.1186/s12887-022-03786-1 (PMC9806818; doi:10.1186/s12887-022-03786-1)
Supplement: Supplementary file 3 — Additional file 3. Health-related quality of life outcomes. [file 12887_2022_3786_MOESM3_ESM.pdf]

Additional file 3

Health-related quality of life outcomes assessed with the Cerebral Palsy Quality of Life Questionnaire for Children (CP QOL-Child), which were completed by the primary caregiver.

Parameter	*n*	Control	12VT	20VT	12VT vs Control	20VT vs Control
**Health**	28	100 (95, 107)	102 (97, 107)	107 (102,112)	3 (-2, 7)	**7 (2, 12)****[**]
**Friends and family**	28	114 (111,118)	114 (110, 117)	113 (109, 116)	0 (-4, 4)	-1 (-6, 3)
**Communication**	28	21 (21, 22)	21 (20, 22)	22 (22, 23)	0 (-1, 1)	1 (-0, 2)
**Pain and bother**	28	34 (31, 36)	33 (31, 36)	32 (30, 35)	0 (-3, 2)	-1 (-4, 2)
**Participation**	28	32 (30, 34)	31 (30, 33)	32 (31, 34)	-1 (-2, 1)	0 (-2, 2)
**Parents' health**	28	27 (26, 29)	28 (26, 29)	28 (26, 29)	0 (-1, 2)	1 (-1, 2)

12VT, assessment after 12 weeks of side-alternating vibration therapy; 20VT, assessment after 20 weeks of side-alternating vibration therapy.
Data at each assessment are the adjusted means and 95% confidence intervals (CI), while differences between assessments are the adjusted mean differences and 95% CI; all values were derived from linear mixed models based on repeated measures, adjusted for the participant's GMFCS level, group allocation (20 Hz / 25Hz), baseline value of the outcome, and the number of days elapsed from baseline.
n is the number of participants at baseline; the number of participants who completed a given assessment is provided in Additional file 1.
**p<0.01 for the difference between 20VT and Control.
